# Genetic, Epidemiological, and Clinical Risk Factors for Perinatal Anxiety and Depression in Dubai: Protocol for a 2-Point Prospective Observational Study

**DOI:** 10.2196/68346

**Published:** 2025-04-29

**Authors:** Zenab Yusuf Tambawala, Nusrat Khan, Shabnam Saquib, Jeyaseelan Lakshmanan, William Atiomo

**Affiliations:** 1 Mohammed Bin Rashid University of Medicine and Health Sciences Dubai United Arab Emirates; 2 Dubai Hospital Dubai United Arab Emirates

**Keywords:** perinatal anxiety, perinatal depression, genetic risk factors, clinical risk factors, depression, anxiety, antenatal depression, postnatal depression, antenatal anxiety, postnatal anxiety

## Abstract

**Background:**

Perinatal anxiety and depression can significantly impact maternal well-being, infant development, and mother-child bonding. There is a relative lack of research on the overall burden of and risk factors for perinatal and postpartum depression and anxiety in the Middle Eastern region.

**Objective:**

We aimed to investigate genetic, epidemiological, and clinical risk factors for anxiety and depression in antenatal and postnatal mothers.

**Methods:**

This study is a 2-point, cross-sectional, observational study of pregnant women at a tertiary care hospital in Dubai, United Arab Emirates. We will evaluate the point prevalence of depression and anxiety with the Edinburgh Postnatal Depression Scale, the Generalized Anxiety Disorder 7 scale, and the Holmes-Rahe Stress Inventory and analyze the risk factors in affected and unaffected women. The women will be evaluated with structured interviews, initially in the antenatal period (between 20 to 26 weeks) and again in the postnatal period (between 6 weeks to 6 months after delivery). Whole-genome sequencing will be conducted to comprehensively map genomes and detect variants associated with depression and anxiety after the initial interview. Social factors such as family characteristics and partner support, as well as lifestyle factors such as exercise, vitamin D intake, and obstetric factors, along with intrapartum and neonatal events affecting maternal mental health, will also be assessed.

**Results:**

We will assess the prevalence of depression, anxiety, stress, and risk factors in the antenatal and postnatal period between July 2025 and June 2026 at Dubai Hospital. The association of genetic, social, and demographic risk factors with depression and anxiety will be compared in women who screen positive for depression and anxiety and those who screen negative.

**Conclusions:**

This research aims to identify genetic variants associated with perinatal anxiety and depression in Middle Eastern women and to develop a comprehensive risk assessment tool for identifying women at high risk for perinatal anxiety and depression.

**International Registered Report Identifier (IRRID):**

PRR1-10.2196/68346

## Introduction

### Background

The World Health Organization’s guide for integrating perinatal mental health into maternal and child health services reflects the global recognition of this crucial health concern [1]. The impact of mental health disorders on the workforce is significant, with 15% of working-age adults worldwide experiencing such conditions. Anxiety and depression alone lead to the loss of 12 billion working days annually [2]. During pregnancy, untreated depression and anxiety in the perinatal and postpartum periods have far-reaching consequences, beyond the economic implications. These conditions are associated with obstetric complications [3], poor birth outcomes, and reduced health-seeking behavior [4] during and after pregnancy [5]. Furthermore, these conditions can impair mother-child bonding [6], with infants of mothers with such conditions at a higher risk for physical, emotional, and behavioral issues [4]. Addressing perinatal mental health is therefore crucial for the well-being of the mother, child, and the wider family unit in both the short and long term.

Previous publications [6-10] on perinatal mental illness in the United Arab Emirates reveal a wide range in reported prevalence, from 10% to 85.6%. Women of Middle Eastern ethnicity (ie, from the United Arab Emirates, Saudi Arabia, Oman, Kuwait, Yemen, Syria, Iraq, Iran, and Jordan) are highly prone to depression (50% prevalence) and anxiety (48.5% prevalence) compared to women from South and Southeast Asia (from India, Pakistan, and the Philippines: 29% prevalence of depression and 25.2% for anxiety), while North African women (from Egypt, Sudan, and Somalia) have a prevalence of 39.6% for depression and 43.4% for anxiety. Globally, rates of perinatal depression and anxiety are estimated at 1 in 10 in high-income countries and 1 in 5 in low- and middle-income countries [1]. Given these increased local rates, factors causing mental health issues in the obstetric population in this region must be addressed as a priority. There are very few systematic reviews or primary studies of good value from the Middle East and North African region to accurately assess the prevalence of these disorders [6]. Table 1 provides a summary of past results from these studies.

An unpublished pilot study conducted at Dubai Hospital in 2024 found that only 0.65% of pregnant patients treated in the Department of Obstetrics and Gynecology had anxiety, depression, or both documented in their diagnoses. This critical gap in identifying and treating perinatal mental health conditions underscores the urgent need for comprehensive research in this field.

**Table 1:** Prevalence of maternal mental health disorders in the Middle East and North African region in past studies.

Country	Study	Year	Sample size, n	EPDS^a^ score	Antenatal depression, %	Postnatal depression, %	Antenatal anxiety, %	Postnatal anxiety, %	Both anxiety and depression, %
Egypt	Saleh et al [11]	2013	120	≥13	—^b^	18	—	—	—
Oman	Al Hinai and Al Hinai [12]	2014	282	≥13	—	12	—	—	—
Oman	Al-Azri et al [13]	2016	959	≥13	24		—	—	—
United Arab Emirates	Alhammadi et al [14]	2017	504	≥10	—	33	—	—	—
Saudi Arabia	Alqahtani et al [15]	2018	575	≥14	27	—	24	—	—
Turkey	Durankuş and Aksu [16]	2022	260	≥13	35	—	—	—	—
Egypt	Khamees et al [17]	2021	120	≥14	44	—	—	—	—
Turkey	Guvenc et al [18]	2021	212	≥13	—	34	—	—	—
United Arab Emirates	Tambawala et al [10]	2023	438	≥10	43	44	44	40	32

^a^EPDS: Edinburgh Postpartum Depression Scale.

^b^Not available.

Several risk factors are thought to increase the risk of poor perinatal mental health [8]. These factors encompass sociodemographic, genetic, lifestyle, clinical, and endocrine risk factors. Risk factors also include depression during the second and third trimesters of pregnancy, the number of children, religion, and the use of formula for feeding. Additionally, factors like educational level, lack of breastfeeding, stressful life events [1], gestational diabetes [19], and employment status after delivery have been found to be of borderline significance [8]. These risk factors are crucial for early identification, underscoring the importance of screening pregnant women during pregnancy and post partum. We therefore aim to evaluate the most relevant sociodemographic risk factors for perinatal anxiety and depression among pregnant women in Dubai. Studying the specific risk factors in our local population will help identify region-specific factors that could allow for more tailored interventions to improve outcomes for mothers and infants in Dubai.

Recent studies have identified multiple genes that contribute to both depression and anxiety, with many genes being common to both conditions [20]. Analysis of genome-wide association studies from the Psychiatric Genomics Consortium database found a very strong correlation between anxiety and depressive disorders and also revealed a complex interplay of multiple genetic variants that underlie these psychiatric disorders. Studies also indicate that variations in genes related to the hypothalamic-pituitary-adrenal axis, serotonin transporters, and other neurotransmitter systems probably increase the risk of anxiety and depression [21-23].

Understanding these genetic risks in greater depth is critical for determining the underlying molecular mechanisms of these disorders, which may lead to the development of more tailored therapeutic strategies. Furthermore, understanding the interplay of genetic and environmental risk factors during the perinatal period should inspire preventive treatments that improve both maternal health and newborn development.

To the best of our knowledge, no previous study has analyzed perinatal mental health by looking at genetic, clinical, and sociodemographic risk factors in the United Arab Emirates. These risk factors need to be identified to prevent, screen, diagnose, and treat anxiety and depression during this vulnerable period.

### Objectives

This study aims to investigate the sociodemographic, genetic, lifestyle, and clinical risk factors associated with anxiety and depression in perinatal and postnatal women in Dubai. (Textbox 1).

The prevalence of anxiety and depression in perinatal and postnatal women in Dubai is higher than global averages and is associated with a unique cluster of sociodemographic, genetic, lifestyle, and clinical risk factors. Cultural factors, traditional family structures, and social expectations could be protective or may hinder access to mental health services in this region. There is a need to assess the prevalence rate of anxiety and depression and associated genetic factors in the Middle Eastern region. As in type 2 diabetes and obesity, genetic susceptibility to psychiatric disorders could be more prevalent in the Middle Eastern region compared to other populations. Understanding these genetic risks in greater detail is crucial for uncovering the underlying biological mechanisms of these disorders, potentially leading to the development of more targeted therapeutic interventions. Moreover, elucidating the interplay between genetic and environmental risk factors during the perinatal period could inform preventive strategies that benefit maternal health and infant development.

**Objectives.**

**Primary objective**

- To investigate the point prevalence of anxiety and depression in antenatal and postnatal mothers using the Edinburgh Postnatal Depression Scale and Generalized Anxiety Disorder 7 scale in Dubai Hospital

**Secondary objectives**

- To identify genetic variants in severe cases of depression and anxiety
- To find correlations among the stress, demographic, social, clinical, and lifestyle factors associated with the mental health of antenatal and postnatal women

## Methods

### Recruitment

**Figure 1:**
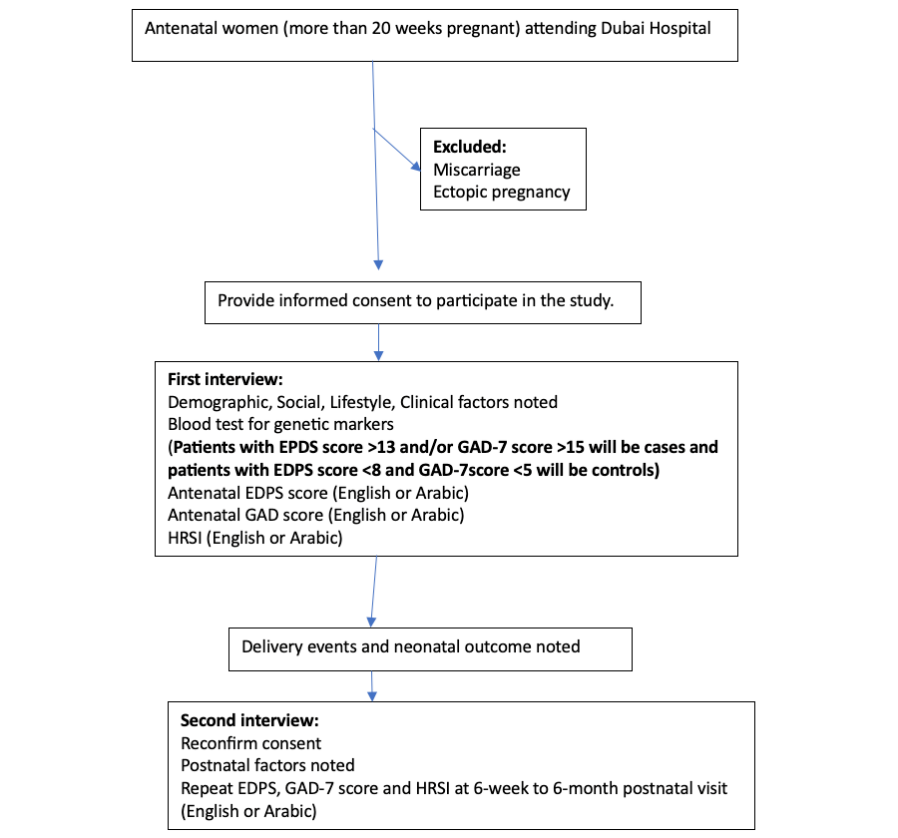
Patient recruitment and study interventions. EPDS: Edinburgh Postnatal Depression Scale; GAD-7: Generalized Anxiety Disorder 7; HRSI: Holmes-Rahe Stress Inventory.

Figure 1 outlines the variables that will be obtained from the participants, with further details provided below.

This will be a cross-sectional observational study conducted at Dubai Hospital, a tertiary center that receives patients from all 7 emirates of the United Arab Emirates. Participants will be pregnant women who, after 20 weeks of gestation, attend antenatal clinics and inpatient admissions at Dubai Hospital. Dubai Hospital caters to Emirati nationals as well as expatriates from mainly South Asia, Southeast Asia, and North Africa, including countries such as India, the Philippines, Egypt, and Sudan. Emirati nationals comprise 65% of the patients and will be the main group in the study. The age range of our participants will be 15 to 50 years.

Participants will be recruited using convenience sampling of pregnant patients attending Dubai Hospital during the study period. Patients who provide detailed informed consent and agree to participate in the study will be interviewed while in the clinic for their antenatal care appointments. A similar study [10] conducted during the COVID-19 pandemic revealed that patients were very willing to discuss their mental health issues confidentially in a safe environment. Women with any preexisting diagnosed psychiatric condition will also be included in our prevalence study, as the association of genetic and other risk factors in these women will be helpful for our results. Exclusion criteria will be the inability to give informed consent, the inability to understand Arabic or English, a lack of willingness to be contacted for follow-up, and a lack of willingness to undergo genetic testing.

### Testing

After an interview, the patient will be directed to give a blood sample for genetic testing. We aim to evaluate 100 patients for genetic risk factors. The first 50 patients with an Edinburgh Postpartum Depression Scale (EPDS) score ≥13 and/or Generalized Anxiety Disorder 7 (GAD-7) score ≥15 will be assigned as cases, and the first 50 patients with an EDPS score <8 and GAD-7 score <5 as controls. The timing of the blood sample collection will coincide with the 24-week glucose tolerance test. This scheduling will prevent any additional discomfort among the pregnant women.

To evaluate genetic factors, we will use a long-read genome sequencing platform. The sequencing data will be analyzed using a Flamingo server, a high-performance computing cluster. This computational infrastructure will enable the robust and efficient processing of the genomic data required for our analysis. The system requires 8 ml to 10 ml of blood and high-molecular-weight DNA. After a long read of whole genome sequencing, established pipelines for genome mapping and variant detection will be used. We will try to identify rare variants that might play a vital role in the etiology of the condition.

### Data Collection

The prevalence of postnatal depression, anxiety, and stress at 6-week to 6-month postnatal visits will be assessed for the same patients whose antenatal EPDS, GAD-7 and Holmes-Rahe Stress Inventory (HRSI) scores were taken. Delivery and postnatal events will also be noted at these visits.

Demographic data, lifestyle factors, and social factors, along with the EPDS, GAD-7, and HRSI scores, will be noted by a trained researcher in a structured interview. Obstetric factors and delivery events will be obtained from the electronic medical records of the patients. Demographic information will include age, nationality of the women and their partners, emirate of residence, BMI, marital status, educational level, occupation, length of working hours, temporary or permanent nature of employment, being a health care worker, and total monthly income. The partners’ education, occupation, consanguinity, military service, and age difference with the participant will also be recorded.

Social factors such as family size (nuclear or extended), loss of a parent in childhood, number of years of marriage, whether the marriage is polygamous, history of intimate partner violence, history of partner mental illness, whether the pregnancy is planned or unplanned, whether there is a support network including the extended family, financial difficulties, loneliness, body image, drug use, alcohol misuse, and chronic illness will be noted. A personal or family history of mental illness will be recorded.

Lifestyle factors will be evaluated, including diet, caffeine intake, sleep disturbance, and vitamin D deficiency, as well as whether the participant exercises at least 30 minutes a day or has sedentary habits. Clinical high-risk factors such as grand multiparity, poor obstetric history, assisted conception, gestational diabetes mellitus, gestational hypertension, cigarette or alcohol use during pregnancy, or any preexisting medical disorders will be mentioned.

Delivery events and postnatal factors such as birth by caesarean section; intrapartum complications; congenital malformations; neonatal intensive care unit admission, with duration of admission; crying, sleeping, or feeding problems of the neonate; gestational age at delivery; husband or family presence at delivery; gender of baby; and baby birth weight will be noted. History of postnatal emotional support from family, poor maternal sleep, and use of maternity leave will be obtained from the postnatal women at the second interview.

### Mental Health Assessment Tools

**Figure 2:**
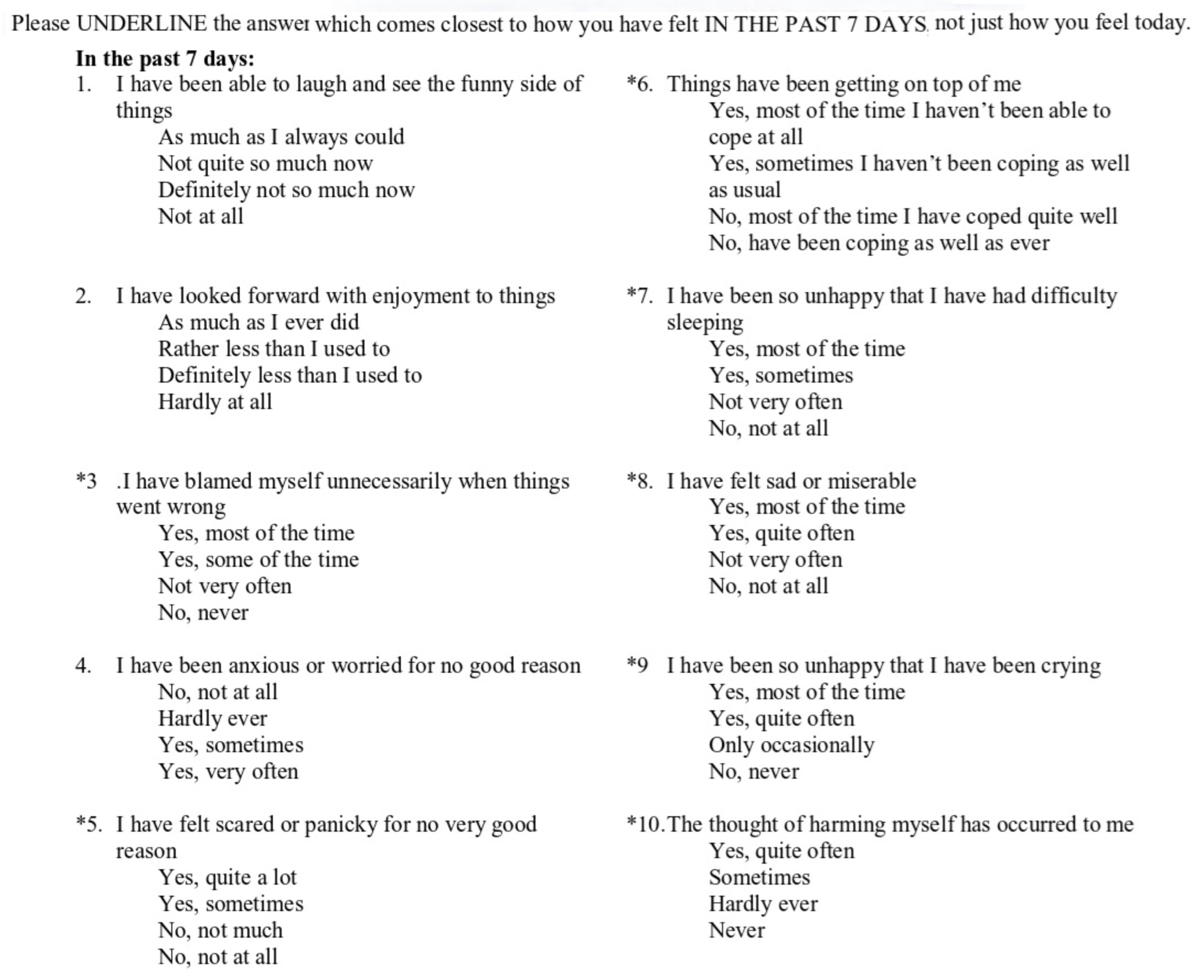
Edinburgh Postpartum Depression Scale (EPDS).

Depression symptoms will be assessed by the EPDS (Figure 2), which is validated for both English and Arabic [7,24,25]. It is a self-reported 10-item questionnaire scored from 0 to 3, with the minimum and maximum total scores ranging from 0 to 30 points. A cutoff of ≥10 points is used for identifying depression and ≥13 for severe depression; a “yes” response to question 10 is used to identify suicidal ideation.

**Figure 3:**
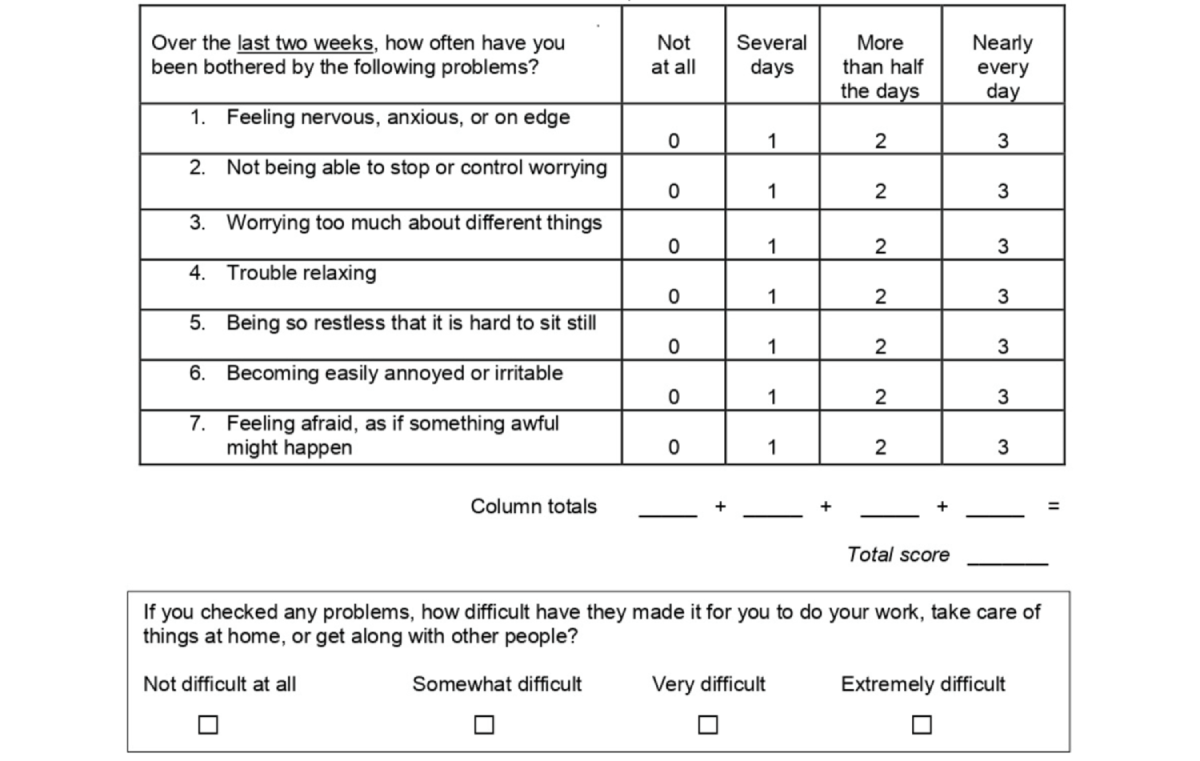
Generalized Anxiety Disorder (GAD-7) questionnaire.

Anxiety symptoms will be assessed using validated English and Arabic-translated versions of the Generalized Anxiety Disorder 7 (GAD-7) questionnaire [26] (Figure 3). This 7-item questionnaire assesses tension, restlessness, and irritability and is scored from 0 to 3 and has an overall score range of 0 to 21. A score ≥5 will be considered to indicate a risk of anxiety and will be used as a cutoff in our study.

**Figure 4:**
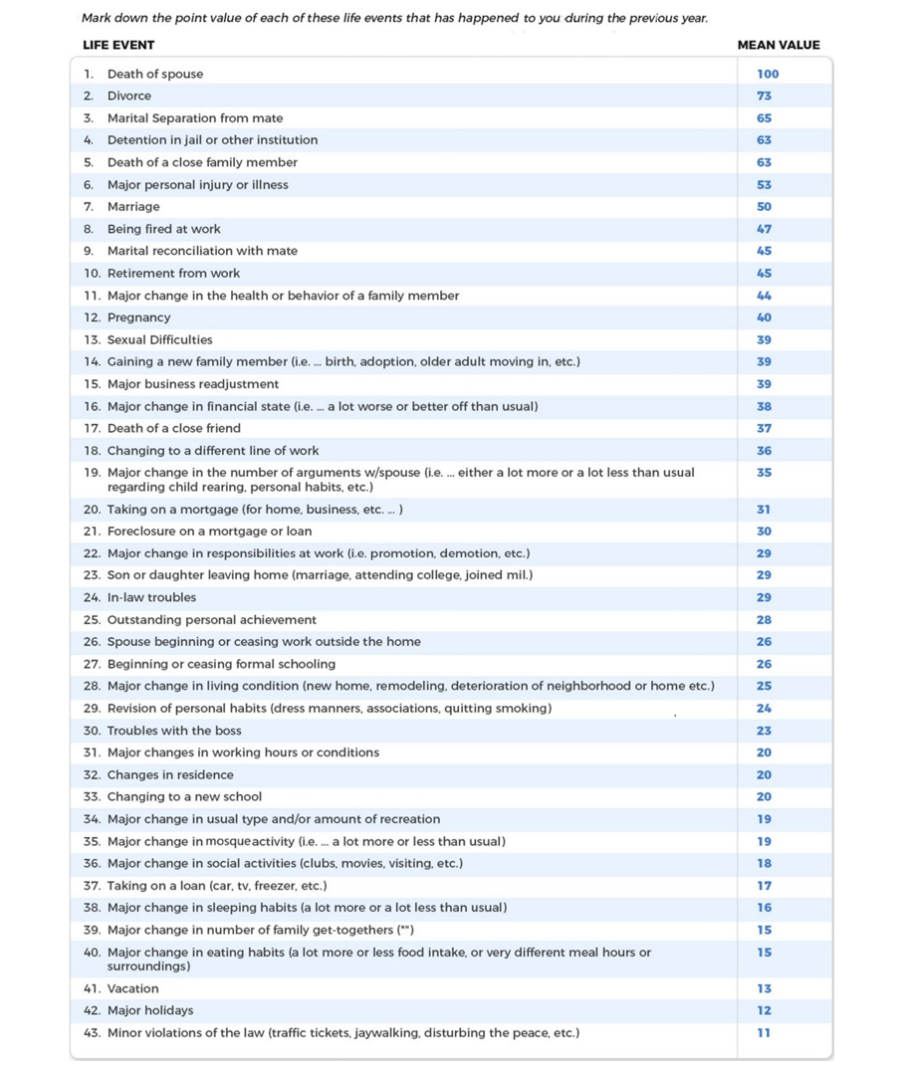
Holmes-Rahe Stress Inventory (HRSI).

The HRSI [27] (Figure 4) will be used to identify the level of stress with life change unit values. The cumulative impact of these life changes can significantly affect one’s health, emphasizing the importance of managing and mitigating stress. A score of 150 points or less indicates a relatively low amount of life change and a low susceptibility to stress-induced health breakdown. A score of 150 to 300 points indicates a 50% chance of a health breakdown in the next 2 years, and 300 points or more implies an 80% chance of a health breakdown in the next 2 years.

### Sample Size

The sample size for this prevalence study was calculated based on an expected prevalence of 33% (expressed as 0.33), as reported in a previous study [14] on postnatal depression in Dubai, a desired precision of 5% (expressed as 0.05), and a 95% confidence level. The required sample size is approximately 350 participants. We will, however, aim to recruit 420 women to allow for a 20% dropout rate. We are confident we will achieve this number given the number of pregnant women seen at Dubai Hospital in one year.

### Statistical Analysis

Data analysis will be done using SPSS Statistics (version 25.0; IBM Corp). Univariate statistics and distribution will be assessed, and continuous variables will be presented as the mean and SD or median and IQR. Categorical variables will be expressed as the frequency and percentage. Initial associations between genetic variants of the serotonin gene (*5-HTTLPR*) and oxytocin gene (*OXTR*) with depression and anxiety will be tested using the *χ*^2^ test. The Fisher exact test will be used to evaluate genotype-phenotype relationships.

The normality of continuous variables will be assessed using the Kolmogorov-Smirnov test. A significance level of *P*<.05 will be used to determine deviation from a normal distribution. Variables found to be nonnormally distributed will be analyzed using appropriate nonparametric statistical methods. To account for potential confounding factors, logistic regression will be used. Linear regression will be applied to continuous outcomes, such as severity scores. The correlations between demographic factors, clinical factors, lifestyle factors, stress, and antenatal EPDS and GAD-7 scores, as well as the correlations between postnatal factors and EPDS and GAD-7 scores of the postnatal patients, will be calculated using Pearson correlations, Kendall rank correlations, and Spearman correlation tests. The Mann-Whitney *U* test will be used for pairs of independent samples and the Kruskal-Wallis test for sets of more than 2 independent demographic variables.

### Ethical Considerations

Informed consent will be obtained from all participants, and they will be provided clarification on the aim of the study and assurance about information confidentiality. Participants can withdraw consent at any point. Women identified as having severe depression or anxiety by the trained interviewer will be referred to our hospital psychologist immediately. Women identified as suicidal (based on their answer to question 10 of the EPDS) will be promptly referred to psychiatry services in accordance with the hospital referral system. Ethical approval will be obtained from the Hospital Ethics Committee and the Dubai Scientific and Research Ethics Committee. The data will be deidentified and no monetary compensation will be given.

## Results

The point prevalence of prenatal and postnatal depression and anxiety will be determined at our institution. The genetic markers associated with depression and anxiety in participants of Middle Eastern ethnicity will be identified, and the relationship between other risk factors and their mental health impact will be investigated in the enrolled patients.

## Discussion

### Comparison With Prior Work

There are only a few past studies on perinatal anxiety and depression in the Middle Eastern region. One study [14] conducted before COVID-19 showed a 33% prevalence of postpartum depression. A comparable study [10] conducted at the same location during the pandemic found a 43.6% prevalence of depression and 42% prevalence of anxiety. However, the risk factors were not assessed in these studies. We discovered just one study [28] that examined the relationship between genetic markers and postnatal depression in Jordanian women. The overall prevalence of maternal mental health problems in Middle East is higher than other regions of the world, and the reasons behind this disparity need to be investigated.

### Limitations

While this study design should provide useful information about perinatal mental health, some limitations must be addressed. The recruitment of participants from a single tertiary care hospital in Dubai may impact the generalizability of our findings. The calculated point prevalence may not accurately reflect the true burden of perinatal mental health problems in the population. The study population may not accurately reflect the larger community, particularly those who do not have access to tertiary health care services.

Measurement constraints include the use of self-reported screening measures like the EPDS and GAD-7. Although validated, these tools are susceptible to reporting bias and may be influenced by cultural variables influencing how women in this region perceive and report mental health symptoms. The targeted method for identifying genetic factors may overlook other relevant genetic variants. Furthermore, the cross-sectional design limits our capacity to assess how these genetic factors interact with environmental cues during the perinatal period.

Despite these limitations, this study is an essential step toward better understanding perinatal mental health in the Middle East, and the data will be useful for future longitudinal and more extensive studies in this field.

### Conclusions

This study is designed to provide a deeper understanding of the multifaceted factors contributing to anxiety and depression in antenatal and postnatal women. By engaging in this research, the clinicians involved will gain expertise in using validated mental health assessment tools like the EPDS and GAD-7 scales and in understanding the implications of genetic causes for depression and anxiety. Furthermore, the study’s comprehensive approach, which includes sociodemographic, lifestyle, and clinical factors, will enable obstetricians and gynecologists to adopt a more holistic and informed approach to patient care. This enriched understanding will enhance the ability to identify at-risk individuals, implement early interventions, and collaborate effectively with mental health professionals.

## Abbreviations

EPDS: Edinburgh Postnatal Depression Scale
GAD-7: Generalized Anxiety Disorder 7
HRSI: Holmes-Rahe Stress Inventory

## Data Availability

The data supporting the findings of this study will be made available from the corresponding author upon reasonable request to any researcher wishing to use them for noncommercial purposes without breaching participant confidentiality. The data will not be publicly available due to privacy and confidentiality concerns.
